# Intramuscular Fat Infiltration and Knee Osteoarthritis: A Systematic Review and Meta‐Analysis

**DOI:** 10.1002/pri.70330

**Published:** 2026-08-26

**Authors:** Lili Yu, Cong Chen, Guancong Zhang, Qiuchen Huang, Miao Ye, Yukiko Makihara, Yusuke Nishida

**Affiliations:** ^1^ Department of Physical Therapy Graduate School of Health Sciences at Narita International University of Health and Welfare Narita City Chiba Japan; ^2^ Department of Physical Therapy China Rehabilitation Research Center Beijing China

**Keywords:** knee osteoarthritis, meta‐analysis, muscle fat infiltration, systematic review

## Abstract

**Background:**

Intramuscular fat infiltration (IMFI) is considered to be closely associated with knee osteoarthritis (KOA); however, its overall association, muscle‐specific involvement, and clinical relevance remain unclear.

**Objective:**

To quantify the association between IMFI and KOA, identify affected muscle groups, and evaluate prognostic value.

**Methods:**

We searched PubMed, Web of Science, Scopus, and Embase up to December 2025 for observational studies. Two reviewers independently screened, extracted data, and assessed bias (NOS/JBI). Pooled standardized mean differences (SMDs) were calculated using random‐effects models, with subgroup and sensitivity analyses.

**Results:**

Seventeen studies were included. IMFI was significantly higher in KOA patients than controls (SMD = 0.63, 95% CI 0.41–0.85, *I* ^2^ = 84.7%). Anatomically, the anterior thigh muscles (rectus femoris, vastus lateralis, vastus medialis) demonstrated a significant pooled effect (SMD = 0.96, 95% CI 0.66–1.25, *I* ^2^ = 3.2%) and showed the strongest pooled association, whereas posterior, medial, and composite measures did not demonstrate significant associations. At the individual muscle level, a single study reported a notable association for the semimembranosus (SMD = 1.25, 95% CI 0.57–1.93); however, this finding is based on limited evidence and requires confirmation in future studies. Subgroup by imaging modality: CT demonstrated a significant association (SMD = 0.59, 95% CI 0.28–0.90), while MRI and ultrasound did not show significant associations. Elevated IMFI was also associated with reduced muscle strength, higher KOA incidence, structural progression, and increased knee replacement risk.

**Conclusion:**

A moderate positive association exists between IMFI and KOA, with notable muscle‐specific heterogeneity. Anterior thigh muscles show the strongest pooled association, whereas individual muscle findings require further confirmation. These findings suggest that IMFI may represent a promising potential marker warranting further prospective investigation for risk stratification, though causality and its role in modifying disease progression remain to be established.

**Abbreviations**
ACR: American College of Rheumatology
BMI: body mass index
CI: confidence interval
CSA: cross‐sectional area
CT: computed tomography
DXA: dual‐energy x‐ray absorptiometry
GBD: global burden of disease
HR: hazard ratio
ICD: international classification of diseases
IMFI: intramuscular fat infiltration
IPD: individual participant data
JBI: Joanna Briggs Institute
K/L: Kellgren–Lawrence
KOA: knee osteoarthritis
MRI: magnetic resonance imaging
NOS: Newcastle‐Ottawa Scale
OR: odds ratio
RCT: randomized controlled trial
REML: restricted maximum likelihood
SMD: standardized mean difference
WOMAC: Western Ontario and McMaster Universities Osteoarthritis Index

## 1 Introduction

Knee osteoarthritis (KOA) is a chronic degenerative joint disease characterized by progressive cartilage loss, subchondral bone remodeling, osteophyte formation, and joint space narrowing. Clinically, it presents with pain, stiffness, and muscle weakness, leading to functional impairment and reduced quality of life (Hunter and Bierma‐Zeinstra 2019). KOA is one of the most prevalent joint diseases globally; the prevalence of radiographic KOA among individuals aged ≥ 60 years ranges from 30% to 50% and continues to rise with population aging and increasing obesity rates (M. Li et al. 2025; Tang et al. 2026). The substantial physical, psychological, and socioeconomic burden positions KOA as a major public health priority (GBD 2021 Osteoarthritis Collaborators 2023).

Traditional pathogenic mechanisms include abnormal joint loading, inflammation, metabolic disturbances, and genetic susceptibility (Hunter and Bierma‐Zeinstra 2019). Among these, quadriceps weakness and atrophy are well‐established risk factors associated with KOA onset and progression (Øiestad et al. 2015; Wang et al. 2012). However, accumulating evidence suggests that changes in muscle quality—particularly intramuscular fat infiltration (IMFI)—may better reflect muscle function than volume loss alone and play an independent role in KOA pathology (Kumar et al. 2014; Mohajer et al. 2022; Taniguchi et al. 2023; Weng et al. 2025).IMFI is defined as abnormal fat deposition within or between muscle fibers, quantifiable by MRI, CT, DXA, ultrasound, or biopsy (Garcia‐Diez et al. 2024). Elevated IMFI compromises contractile strength and fatigue resistance, and may be linked to cartilage degeneration and synovitis through pro‐inflammatory adipokines (e.g., leptin, TNF‐α), oxidative stress, and altered joint mechanics (Biltz et al. 2020; Gonzalez et al. 2021; Kawai et al. 2021; Manatrakul et al. 2024). Thus, IMFI reflects a pathological shift of muscle from contractile to adipose tissue.

Recent studies have shown that IMFI in the quadriceps of KOA‐affected limbs is elevated and correlates with worse radiographic grading, WOMAC scores, and structural progression (Kumar et al. 2014; Weng et al. 2025). Pedroso et al. (2019) conducted a meta‐analysis of 7 cross‐sectional studies comparing IMFI between KOA patients and controls, but did not examine muscle‐specific patterns, high‐ versus low‐IMFI comparisons, or prognostic outcomes. Moreover, existing studies show considerable heterogeneity in effect sizes, muscle regions, measurement techniques, and IMFI quantification thresholds (Manatrakul et al. 2024; Mohajer et al. 2022; Raynauld et al. 2015). Compared to Pedroso et al. (2019), our review provides an updated and more comprehensive analysis including 17 studies, muscle‐specific subgroup analyses, evaluation of imaging modalities, and synthesis of prognostic outcomes.

Therefore, this updated systematic review and meta‐analysis aimed to: (1) quantify the overall association between IMFI and KOA; (2) identify the most relevant muscle groups; (3) assess the influence of imaging modalities on effect sizes and heterogeneity; and (4) synthesize the prognostic value of IMFI for muscle strength loss, KOA onset, structural progression, symptom worsening, and knee replacement risk. The primary outcome was the pooled standardized mean difference (SMD) in IMFI between KOA patients and controls. These analyses were intended to provide a broader evidence base for future risk stratification and early intervention hypotheses in KOA.

## 2 Methods

### 2.1 Literature Search Strategy

We systematically searched PubMed, Web of Science, Scopus, and Embase from inception to December 15, 2025. Search strategies were developed using a combination of Medical Subject Headings (MeSH) and free‐text terms. The English search string comprised the following terms: (“muscle fat infiltration” OR “intramuscular fat” OR “myosteatosis” OR “muscle adipose tissue”) AND (“knee osteoarthritis” OR “knee OA” OR “gonarthrosis”). The detailed search strategies and preliminary results for each database are provided in Supporting Information S2: Table S1. No restrictions were imposed on language or study type. Two independent researchers performed all literature searches; discrepancies were resolved through discussion or by consultation with a third researcher. This study was prospectively registered on the PROSPERO platform (registration No. CRD420251138472). The analysis adhered to the pre‐specified protocol with no deviations.

### 2.2 Inclusion and Exclusion Criteria

We screened the relevant literature using predefined eligibility criteria. Inclusion criteria: (1) participants were adults diagnosed with KOA (Kellgren‐Lawrence Grade ≥ 2 or meeting ACR criteria); (2) the exposure of interest was quantitatively assessed IMFI using MRI, CT, DXA, ultrasound, or muscle biopsy; (3) studies included one of the following comparisons: high‐ versus low‐IMFI, KOA versus healthy/non‐KOA controls, affected versus unaffected limbs, or KOA progression versus non‐progression; (4) studies reported at least one KOA‐related outcome, including radiographic grading, joint structural damage, symptom scores (WOMAC), muscle strength, or knee replacement risk; (5) study designs included prospective cohort, cross‐sectional, case‐control, or longitudinal analyses of randomized controlled trials. Exclusion criteria: (1) non‐original articles (e.g., reviews, systematic reviews, case reports, or conference abstracts); (2) animal or non‐human studies; (3) studies not involving KOA or not measuring IMFI; (4) studies with non‐compliant designs (e.g., lacking a control group, or reporting only muscle volume or strength without IMFI data); (5) studies with unavailable full texts or insufficient effect‐size data; (6) studies with control groups that did not meet requirements (e.g., presence of comorbidities potentially confounding IMFI measurements).

### 2.3 Literature Screening and Data Extraction

All retrieved records were imported into EndNote 21 for deduplication and organization. Two independent reviewers screened the records following the PRISMA guidelines. Inter‐rater agreement was assessed using Cohen's κ statistic, which was 0.89 for title/abstract screening and 0.92 for full‐text screening, indicating excellent agreement. First, obviously irrelevant studies were excluded by screening titles and abstracts; subsequently, full‐text articles were retrieved for secondary screening to determine final eligibility. When multiple publications arose from the same cohort, the version with the longest follow‐up or the most comprehensive data was included. A pre‐designed data extraction form was used to collect the following information: first author, publication year, country, study design, sample size, age, sex ratio, BMI, KOA diagnostic criteria, KL Grade distribution, IMFI measurement method, muscle sites assessed, IMFI quantification metrics, definitions of high‐ and low‐score groups, effect sizes, and corresponding 95% confidence intervals. When data in a publication were incomplete or ambiguous, the corresponding authors were contacted via email to obtain supplementary information. All data extraction was performed independently by two reviewers; any discrepancies were resolved through discussion or by adjudication from a third reviewer.

### 2.4 Bias Risk Assessment

The Newcastle‐Ottawa Scale (NOS) was used to assess the methodological quality of the included prospective cohort studies (including longitudinal analyses of randomized controlled trials) (Wells et al. 2000). The NOS comprises 9 items and evaluates studies across three domains: selection of study populations, comparability between groups, and ascertainment of outcomes. Scores of 7–9 indicated high quality, 5–6 moderate quality, and ≤ 4 low quality. The quality of the included cross‐sectional studies was assessed using the Cross‐Sectional Study Evaluation Tool developed by the Joanna Briggs Institute (JBI) (2017). This tool comprises 8 items, each scored 1 point, yielding a total possible score of 8. Scores of 7–8 indicated high quality, 5‐6 moderate quality, and ≤ 4 low quality. The risk‐of‐bias assessment was performed independently by two reviewers; disagreements were resolved through discussion, and a third reviewer was consulted when consensus could not be reached.

### 2.5 Statistical Analysis

This meta‐analysis included only studies that compared a KOA group with a healthy or non‐KOA control group. Although the terminology describing knee osteoarthritis varied across studies (e.g., OA, ROA, KOA), all diagnoses were based on a KL Grade ≥ 2 or the ACR criteria; for the purposes of this analysis, all cases were uniformly classified as KOA (Altman et al. 1986; Kellgren and Lawrence 1957). We extracted sample sizes, means, and standard deviations for each group and calculated the standardized mean difference (SMD). For measures with inverse directionality (e.g., CT attenuation values, where higher values indicate lower fat infiltration), we reversed the sign of the SMD to ensure that the effect direction consistently reflected higher fat infiltration or more severe outcomes in the KOA group.

SMDs were pooled using a random‐effects model, with 95% confidence intervals and *p*‐values calculated using the Hartung‐Knapp correction (Hartung and Knapp 2001). To ensure the independence of effect sizes, the following approach was adopted. For subgroup analyses where a single study contributed multiple effect sizes to the same subgroup (e.g., multiple anterior thigh muscles from the same cohort), we first pooled these within‐study effects using a fixed‐effect model to generate a single study‐level estimate before inclusion in the main random‐effects meta‐analysis. For individual muscle‐level analyses, each study contributed only one effect size per muscle group. This procedure ensures that no single cohort disproportionately influences the pooled estimates. Heterogeneity was quantified using the *I* ^2^ statistic, with values of < 25%, 25%–75%, and > 75% indicating low, moderate, and high heterogeneity, respectively (Huedo‐Medina et al. 2006). When *I* ^2^ exceeded 50%, subgroup and sensitivity analyses were performed to explore potential sources of heterogeneity. Subgroup analyses were conducted according to anatomical region (anterior, posterior, medial, and mixed composite measures), individual muscles (e.g., semimembranosus, semitendinosus), and IMFI assessment modality (CT, ultrasound, or MRI). Sensitivity analysis was performed using a stepwise exclusion approach to evaluate the robustness of the pooled effect size to individual studies. Publication bias was assessed qualitatively via funnel plots and quantitatively using Egger's regression test, with *p* < 0.05 indicating the presence of publication bias (Egger et al. 1997). Given the substantial heterogeneity (*I* ^2^ = 84.7%), we explored potential sources through subgroup and sensitivity analyses. The high heterogeneity likely reflects the substantial clinical and methodological diversity across the included studies, including differences in study design (cross‐sectional vs. prospective), participant characteristics (age, BMI, KOA severity), IMFI measurement techniques (CT, MRI, ultrasound), quantification metrics, and muscle groups assessed. No meta‐regression was performed due to the limited number of studies (typically, at least 10 studies per moderator are recommended) and the risk of overfitting.

For other comparisons (e.g., high‐ vs. Low‐IMFI, affected vs. unaffected limbs) and prognostic outcomes, we calculated effect sizes for individual studies but did not pool them quantitatively; these results were summarized descriptively. Given the substantial number of subgroup analyses performed, these results should be considered exploratory, and no adjustment was made for multiple testing. Findings from subgroups with limited studies (e.g., < 3 studies) should be interpreted with particular caution and are hypothesis‐generating rather than confirmatory. All statistical analyses were performed using R software (version 4.5.1) with the metapackage.

## 3 Results

### 3.1 Literature Search and Screening

**FIGURE 1:**
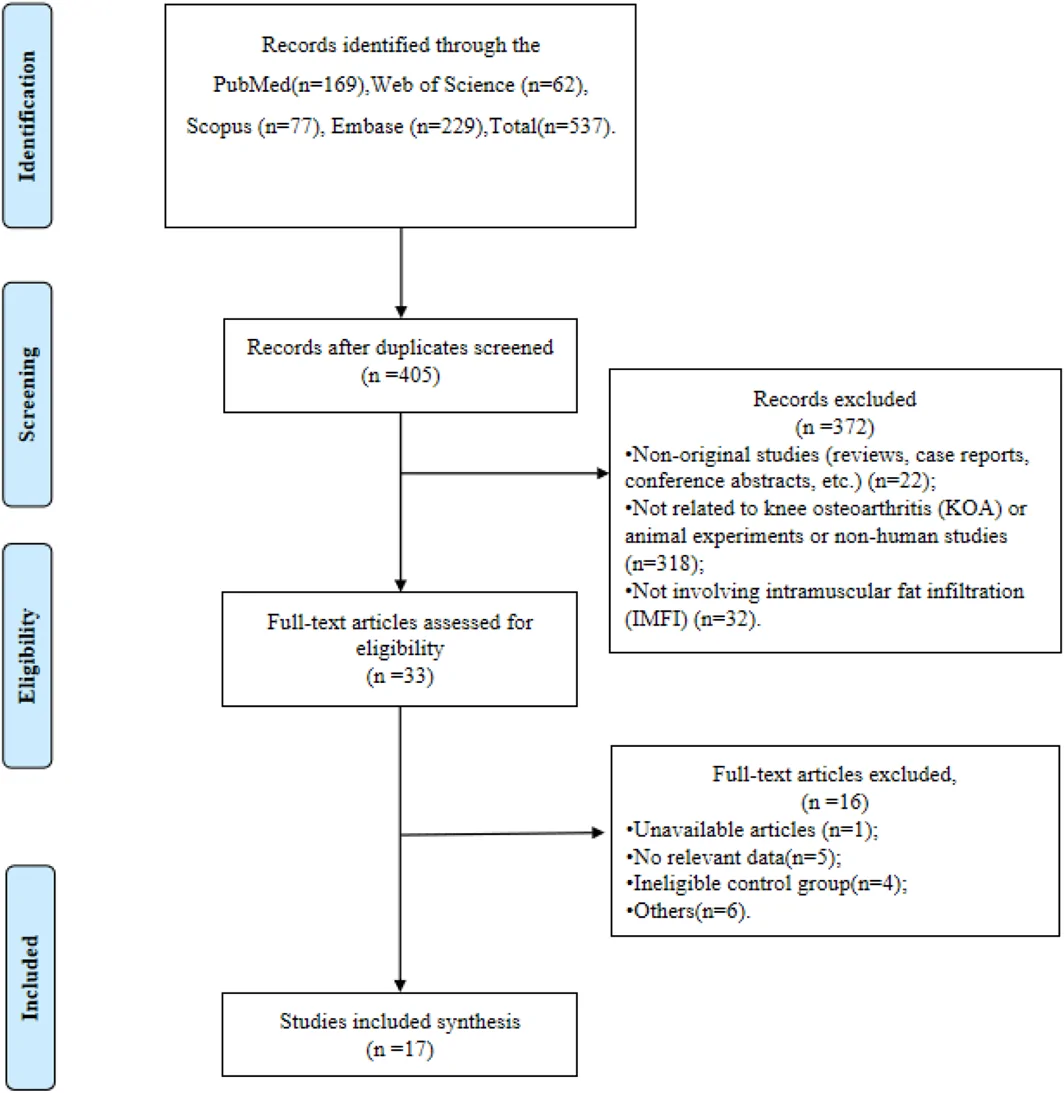
Flowchart of the literature screening process.

A systematic search of the four databases—PubMed, Web of Science, Scopus, and Embase—up to December 15, 2025, identified 537 potentially relevant records. After importing all records into EndNote and removing duplicates, 405 records remained. During the initial screening (title and abstract review), 372 records were excluded. Of these, 22 were non‐original articles (e.g., reviews, case reports, conference abstracts), 318 were unrelated to KOA or involved animal studies, and 32 did not include IMFI measurements. The remaining 33 records proceeded to the second‐round (full‐text) screening. After full‐text review, 16 records were excluded: 1 due to unavailable full text; 5 for insufficient effect‐size data; 4 because the control group did not meet eligibility criteria; and 6 for other reasons (e.g., duplicate publication). Ultimately, 17 records met the eligibility criteria and were included in the final analysis. The screening process and reasons for exclusion are presented in the PRISMA flow diagram (Figure 1). Among the 17 included studies, 7 were prospective cohort studies, 9 were cross‐sectional studies, and 1 was a longitudinal analysis of a randomized controlled trial (RCT).

### 3.2 Study Characteristics

A total of 17 studies, published between 2012 and 2025, were included. The majority originated from the United States (*n* = 6), Canada (*n* = 3), Japan (*n* = 3), and the United Kingdom (*n* = 2). Sample sizes ranged from 69 to 24,224 participants, with the largest cohort reported by Weng et al. The mean participant age ranged from 45.2 to 76.1 years, with the majority being middle‐aged or older adults (> 60 years). The proportion of female participants exceeded 50% in most studies (9/17), and some studies included exclusively female populations (100%). Mean BMI ranged from 20.8 to 31.8 kg/m^2^, with most participants classified as overweight or obese. KOA was primarily diagnosed using the Kellgren‐Lawrence (K/L) grading system (≥ Grade 2), with some studies additionally applying the ACR criteria; one study used ICD‐9/10 codes. The K/L scale ranges from Grade 0 to 4, and the included studies covered a spectrum from early‐stage to severe KOA. IMFI was assessed using MRI, CT, DXA, ultrasound, or muscle biopsy. The primary measurement sites included the anterior thigh muscles (rectus femoris, vastus lateralis, and vastus medialis), followed by the posterior group (semitendinosus, semimembranosus, and biceps femoris long head), the medial group (sartorius, gracilis, adductor magnus/longus), gluteal muscles, and composite indices (e.g., total thigh muscle mass, anterior thigh composite, and total intramuscular fat area). Quantitative metrics included fat fraction (%), fat area/volume, CT attenuation values, ultrasound echogenicity, and muscle volume/cross‐sectional area (CSA), among others. High‐ versus low‐IMFI group definitions varied across studies and included the following comparisons: KOA onset/progression versus non‐progression; KOA versus control; affected versus unaffected limbs; IMFI quartiles; median or pre‐specified thresholds; and continuous variables (per 1% increase). Detailed study characteristics are presented in Table 1 and Supporting Information S2: Table S2.

**TABLE 1:** Basic characteristics of the included studies.

Author, year	Country	Research design	Sample size	Age (years, mean ± SD)	Women (%)	BMI (kg/m^2^, mean ± SD)	KOA diagnostic criteria	KL classification distribution
Beattie et al. (2012)	Canada	Prospective cohort	86	62.7 ± 7.3	100	27.3 ± 5.1	K/L	0–1 versus 2–4
Blumenfeld et al. (2013)	UK	Prospective cohort	909	53.5–55.5	100	24.7–26.4	K/L	0–1 versus ≥ 2
Conroy et al. (2012)	United States	Cross‐sectional	858	73.5 ± 2.9	58	27.9 ± 4.8	K/L ≥ 2	\
Distefano et al. (2024)	United States	Cross‐sectional	655	76.1 ± 4.9	57	27.5 ± 4.4	K/L	0–1, 2, 3–4
Jasinevicius et al. (2024)	Brazil	Cross‐sectional	80	45.3 ± 2.7	\	26.8 ± 2.7	K/L II/III	II or III
Kemnitz et al. (2018)	Multilateral	Prospective cohort	739	64 ± 9	1	28.8 ± 5.9	K/L ≤ 3	0–3
Kitamura et al. (2025)	Japan	Cross‐sectional	77	73.3 ± 7.7	85.7	26.2 ± 3.8	K/L	III, IV
Kumar et al. (2021)	United States	Prospective cohort	69	53.3 ± 10.1	55.2	24.3 ± 3.3	K/L ≥ 2	0–4
Lee et al. (2025)	Korea	Cross‐sectional	89	73 ± 7.8	\	25.6 ± 4.0	K/L ≥ 2	KL 2–4
J. Li et al. (2024)	China	Cross‐sectional	80	66.8 ± 3.6	67.5	24.1 ± 2.4	ACR + K/L ≥ 2	II, III, IV
Manatrakul et al. (2024)	United States	Cross‐sectional	100	62.1 ± 11.1	42	27.2 ± 4.0	K/L ≥ 2	0–4
Mohajer et al. (2023) (DM)	United States	Prospective cohort	698	64 ± 8	60	31.8 ± 4.7	K/L ≥ 2	KL 2–4
Mohajer et al. (2022) (KOA)	United States	Prospective cohort	4634	62 ± 9	56	29.0 ± 4.5	K/L ≥ 2	KL 2–4
Raynauld et al. (2015)	Canada	RCT longitudinal	143	61.0 ± 9.0	67.6	31.6 ± 5.7	K/L ≥ 2	KL 2–3
Taniguchi et al. (2023)	Japan	Cross‐sectional	50	73.5 ± 6.6	46	20.8 ± 2.4	Early‐stage KOA	KL 0–1
Teoli et al. (2022)	Canada	Cross‐sectional	63	58.9 ± 7.6	60.3	27.5 ± 5.5	ACR + K/L ≥ 2	KL 1–4
Weng et al. (2025)	UK	Prospective cohort	24,224	62.95 ± 7.52	52.3	26.61 ± 4.23	ICD‐9/10	\

*Note:* Early KOA is defined as K/L ≤ 1 with knee symptoms.

Abbreviations: \, not reported; ACR, American College of Rheumatology; K/L, Kellgren‐Lawrence grading.

### 3.3 Risk of Bias Assessment

Of the eight prospective cohort studies, six were of high quality and two were of moderate quality. Among the nine cross‐sectional studies, seven were of high quality and two were of moderate quality. Overall, of the 17 included studies, 13 (76.5%) were of high quality and 4 (23.5%) were of moderate quality; no studies were rated as low quality. Thus, the overall risk of bias was considered low based on the NOS and JBI assessments (Supporting Information S2: Table S3).

### 3.4 IMFI and KOA Association

**FIGURE 2:**
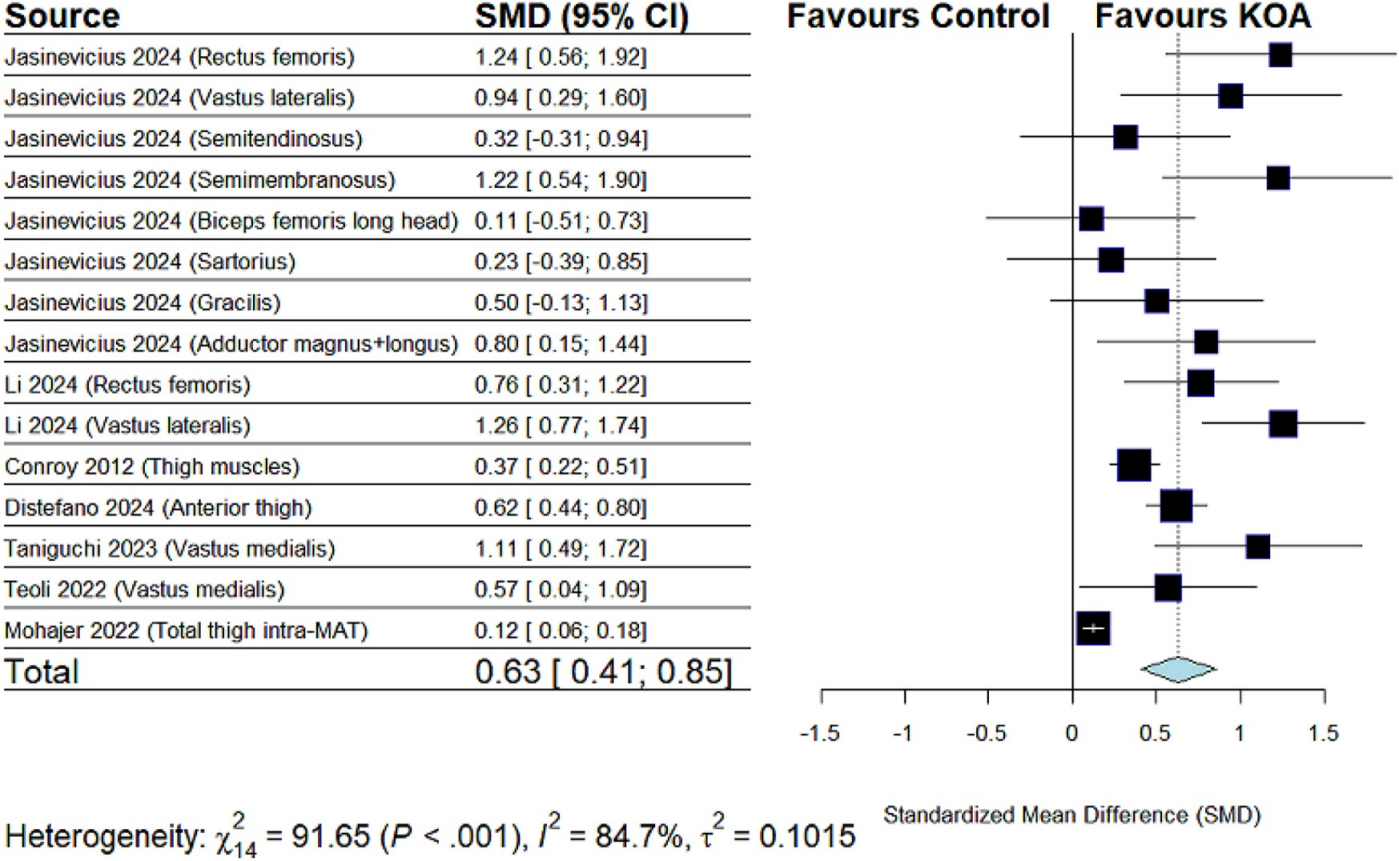
Forest plot of overall effects. Pooled SMD comparing IMFI between KOA patients and healthy/non‐KOA controls.

**FIGURE 3:**
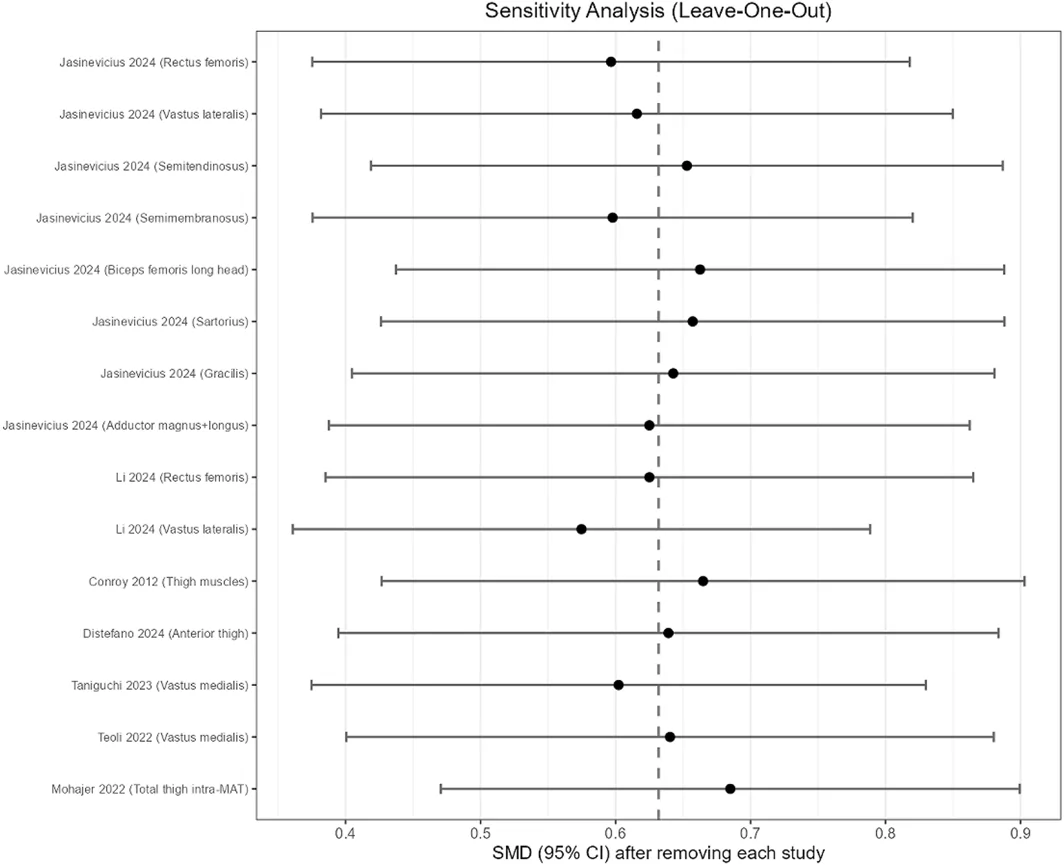
Forest plot of the sensitivity analysis of the overall effect of the association between IMFI and KOA.

**FIGURE 4:**
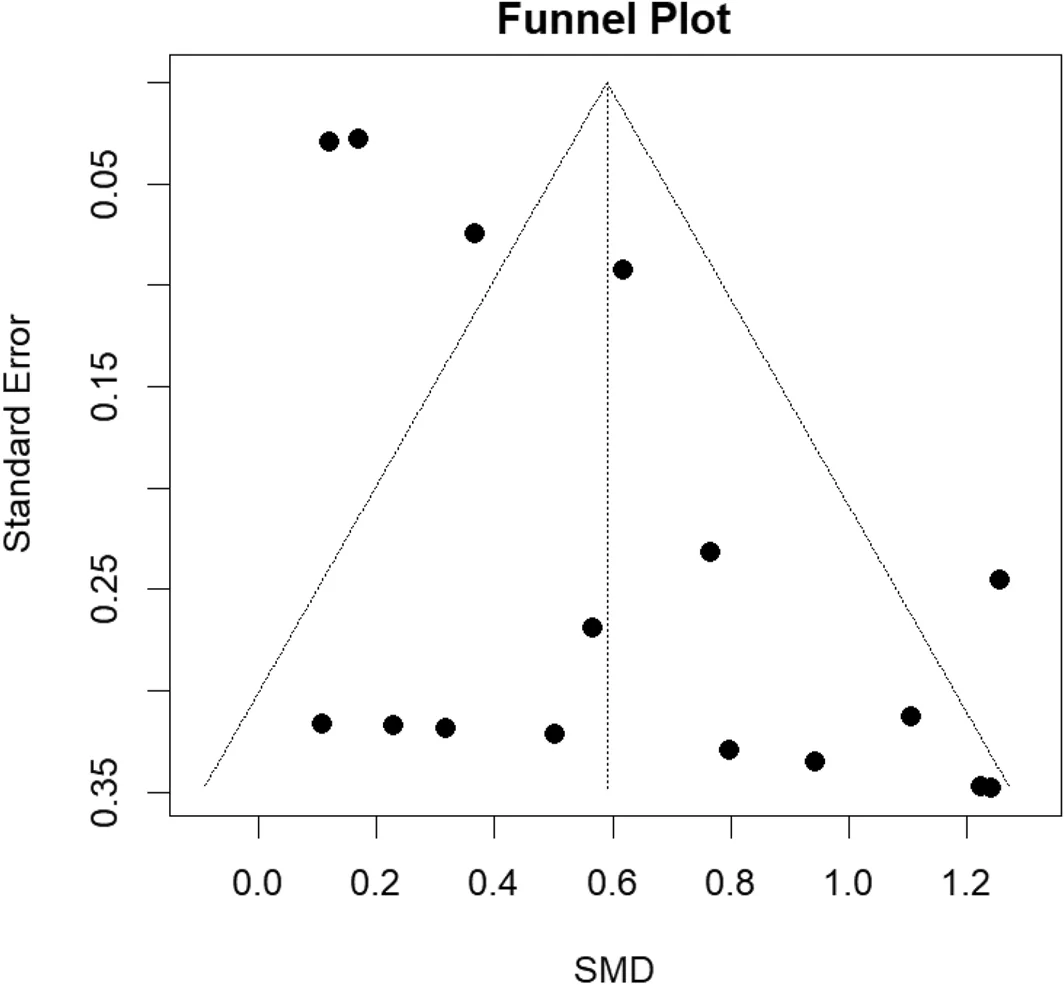
Funnel plot of the overall effect of the association between IMFI and KOA.

Based on comparisons between the KOA and healthy/non‐KOA control groups, a total of 17 studies provided extractable SMDs. A random‐effects meta‐analysis demonstrated that IMFI levels were significantly higher in the KOA group than in controls, with a pooled SMD of 0.63 (95% CI: 0.41–0.85). Substantial heterogeneity was observed across studies (*I* ^2^ = 84.7%, *τ* ^2^ = 0.1015, 95% CI for τ^2^: 0.048–0.312; prediction interval for the true effect in a future study: −0.21 to 1.47; *p* < 0.001), justifying the use of a random‐effects model (Figure 2). Sensitivity analysis revealed that the pooled SMD remained positive after the sequential exclusion of any single study, indicating good robustness of the findings (Figure 3). The funnel plot was largely symmetrical, and Egger's test (*p* = 0.327) provided no evidence of significant publication bias. However, with only 17 studies, the statistical power of Egger's test is limited, and the absence of statistically significant evidence of publication bias does not exclude its presence (Figure 4).

To explore the sources of the substantial overall heterogeneity (*I* ^2^ = 84.7%), we first performed a subgroup analysis by anatomical region (Supporting Information S2: Figure S1), categorizing the measured muscles into four subgroups: anterior, posterior, medial, and mixed composite measures. For the anterior muscle group (rectus femoris, vastus lateralis, and vastus medialis), the pooled SMD was 0.96 (95% CI: 0.66–1.25), demonstrating a moderate positive association, with extremely low within‐subgroup heterogeneity (*I* ^2^ = 3.2%). For the posterior group (semitendinosus, semimembranosus, and biceps femoris long head), the pooled SMD was 0.54 (95% CI: −0.92–2.00), which did not demonstrate a significant association, with moderate within‐subgroup heterogeneity (*I* ^2^ = 67.7%). For the medial group (sartorius, gracilis, and adductor magnus/longus), the pooled SMD was 0.50 (95% CI: −0.20–1.21), which showed no evidence of a significant association, with no within‐subgroup heterogeneity (*I* ^2^ = 0%). For the composite measures (total thigh muscle, anterior thigh composite, and total intramuscular fat area), the pooled SMD was 0.36 (95% CI: −0.26–0.98), which did not reach statistical significance, with extremely high within‐subgroup heterogeneity (*I* ^2^ = 93.8%). The test for subgroup differences was statistically significant (*χ* ^2^ = 11.96, *p* = 0.008), indicating that the strength of the association between IMFI and KOA varied across anatomical regions, with the anterior muscle group emerging as the primary driver of the overall effect.

To further identify the specific muscles most closely associated with KOA, we performed additional subgroup analyses at the individual muscle level. For muscles with multiple available studies (rectus femoris, vastus lateralis, and vastus medialis), none of the pooled estimates reached statistical significance. For muscles examined in only a single study, significant positive associations were observed for the semimembranosus and adductor magnus/longus (Table 2); however, these findings are based on single studies and should be considered preliminary. Significant effects were also found for composite measures including total thigh muscles, anterior thigh, and total intramuscular fat area. In contrast, effect sizes for the semitendinosus, biceps femoris long head, sartorius, and gracilis did not reach statistical significance. In summary, at the individual muscle level, the strongest effect sizes were observed for the semimembranosus and adductor magnus/longus, although these estimates were derived from single studies and should be interpreted as preliminary findings requiring replication in larger, independent cohorts (Table 2).

**TABLE 2:** Effect sizes for the association between muscle fat infiltration and knee osteoarthritis (stratified by muscle region).

Muscle groups	Research (Author, year)	SMD (95% CI)	Heterogeneity *I* ^2^ (%)
Composite/summary measures			
Rectus femoris	Jasinevicius et al. (2024), Li et al. (2024)	0.95 (−2.07, 3.98)	29.0
Vastus lateralis	Jasinevicius et al. (2024), Li et al. (2024)	1.16 (−0.70, 3.02)	0.0
Vastus medialis	Taniguchi et al. (2023), Teoli et al. (2022)	0.83 (−2.65, 4.30)	43.6
Individual muscle groups			
Semitendinosus	Jasinevicius et al. (2024)	0.32 (−0.30, 0.95)	–
Semimembranosus	Jasinevicius et al. (2024)	1.25 (0.57, 1.93)	–
Biceps femoris long head	Jasinevicius et al. (2024)	0.11 (−0.51, 0.73)	–
Sartorius	Jasinevicius et al. (2024)	0.23 (−0.39, 0.86)	–
Gracilis	Jasinevicius et al. (2024)	0.51 (−0.12, 1.14)	–
Adductor magnus + longus	Jasinevicius et al. (2024)	0.81 (0.17, 1.46)	–
Thigh muscles	Conroy et al. (2012)	0.37 (0.22, 0.51)	–
Anterior thigh	Distefano et al. (2024)	0.62 (0.44, 0.80)	–
Total thigh intra‐MAT	Mohajer et al. (2022)	0.12 (0.06, 0.18)	–

*Note:* The pooled effect was calculated using a random‐effects model (with Hartung‐Knapp correction); “–” indicates a single study, for which *I* ^2^ cannot be calculated. Estimates for individual muscles are derived from a limited number of studies (mostly single studies) and should be interpreted as preliminary.

To further evaluate the impact of measurement modality on effect sizes, we performed subgroup analyses stratified by the IMFI assessment method (CT, ultrasound, and MRI) (Supporting Information S2: Figure S2). For the CT subgroup (*k* = 9 effect sizes), the pooled SMD was 0.59 (95% CI: 0.28–0.90), demonstrating a significant positive association, with moderate within‐subgroup heterogeneity (*I* ^2^ = 51.5%). For the ultrasound subgroup (*k* = 2 effect sizes; rectus femoris and vastus lateralis from J. Li et al. 2024), the pooled SMD was 1.00 (95% CI: −2.12–4.12), which did not show a significant association, with moderate within‐subgroup heterogeneity (*I* ^2^ = 52.7%). For the MRI subgroup (*k* = 4 effect sizes), the pooled SMD was 0.54 (95% CI: −0.09–1.16), which did not show a significant association, with very high within‐subgroup heterogeneity (*I* ^2^ = 91.9%). The test for subgroup differences was not statistically significant (*χ* ^2^ = 2.63, *p* = 0.27), suggesting that measurement modality was not the primary source of heterogeneity in this analysis. Overall, the CT subgroup yielded a significant pooled estimate with moderate heterogeneity, suggesting that CT attenuation provides a relatively standardized measure across studies. In contrast, the MRI subgroup showed a non‐significant pooled estimate with very high heterogeneity (*I* ^2^ = 91.9%), which is likely attributable to the limited number of studies and substantial methodological variability across MRI protocols (e.g., T1‐weighted, Dixon, IDEAL sequences) and quantification approaches. The ultrasound subgroup included only two effect sizes from a single study, precluding meaningful inference. These findings should not be interpreted as evidence of CT's superiority over MRI, but rather as reflecting differences in methodological standardization and statistical power across modalities. It is important to note that MRI, CT, and ultrasound quantify IMFI using different physical principles and are not directly interchangeable.

### 3.5 Other Outcome Measures

Due to substantial heterogeneity in study designs, outcome definitions, and comparison groups, the following outcomes were not pooled quantitatively but are summarized narratively. These findings should be considered exploratory and hypothesis‐generating, and they do not carry the same evidential weight as the primary pooled SMD from the main meta‐analysis.

#### 3.5.1 IMFI and Muscle Strength/Function

Five of the included studies reported associations between IMFI and muscle strength or functional measures. Conroy et al. (2012) reported that the RKOA group had significantly lower specific torque than the non‐RKOA group (SMD = 0.39, 95% CI: 0.24–0.53). Distefano et al. (2024) found that patients with KL Grade 3‐4 had lower peak knee extensor power than those with KL Grade 0–1 (SMD = 0.39, 95% CI: 0.21–0.57). Weng et al. (2025) showed that grip strength in the IMFI Q4 group was significantly lower than in the Q1 group (SMD = 1.01, 95% CI: 0.97–1.05). Collectively, these findings consistently indicate that patients with higher IMFI or more severe KOA experience a greater decline in muscle strength.

Taniguchi et al. (2023) reported no significant difference in total quadriceps volume between the early‐stage KOA group and healthy controls (SMD = 0.14, 95% CI: −0.43–0.71). Similarly, Teoli et al. (2022) found no significant difference in the cross‐sectional area of the vastus medialis between the KOA group and healthy controls (SMD = −0.23, 95% CI: −0.75–0.29). However, Mohajer et al. (2022) observed that total thigh CSA was slightly higher in the KOA group than in the non‐KOA group (SMD = 0.08, 95% CI: 0.02–0.13), suggesting inconsistent trends across muscle volume measures.

J. Li et al. (2024) used shear‐wave elastography and muscle tone measurements and found that, on the affected side of KOA patients, the shear modulus of the rectus femoris (SMD = −0.57, 95% CI: −0.91 to −0.24), vastus medialis (SMD = −0.63, 95% CI: −0.97 to −0.29), and vastus lateralis (SMD = −0.72, 95% CI: −1.07 to −0.37) were all significantly lower than on the unaffected side. In contrast, rectus femoris tension (SMD = 0.48, 95% CI: 0.04–0.93) and vastus lateralis stiffness (SMD = 0.53, 95% CI: 0.08–0.97) were significantly higher on the affected side. These findings indicate reduced muscle elasticity and increased muscle tension and stiffness in the affected limbs of KOA patients.

#### 3.5.2 Affected Versus Unaffected Limbs

J. Li et al. (2024) reported that echogenicity of the rectus femoris (SMD = 0.43, 95% CI: 0.11–0.76) and vastus lateralis (SMD = 0.46, 95% CI: 0.13–0.79) was significantly higher on the affected side than on the unaffected side, indicating more severe fatty infiltration in the affected‐limb muscles.

#### 3.5.3 IMFI, KOA Incidence, and Structural Progression

Weng et al. (2025), using data from a large UK Biobank cohort (*n* = 24,224), found that the incidence of knee OA was significantly higher in the upper thigh IMFI Q4 group than in the Q1 group (HR = 2.34, 95% CI: 1.53–3.59). For posterior thigh IMFI, the incidence of knee OA was also elevated in the Q4 group but did not reach statistical significance (HR = 1.26, 95% CI: 0.86–1.84). However, the incidence of hip OA was significantly elevated in both the anterior and posterior thigh Q4 groups (HR = 1.62 and 1.78, respectively). Kumar et al. (2021) reported that each 1% increase in quadriceps intramuscular fat fraction was associated with an 113% increase in the risk of knee MRI lesion progression (cartilage, meniscus, and bone marrow lesions) (OR = 2.13, 95% CI: 1.09–4.15). Similarly, each 1% increase in medial thigh muscle IMFI was associated with a 105% increase in risk (OR = 2.05, 95% CI: 1.25–3.36).

#### 3.5.4 IMFI, Symptom Worsening, and Knee Replacement Risk

Mohajer et al. (2022) found that KOA patients with comorbid diabetes (and higher levels of myosteatosis) had a higher risk of developing unacceptable symptom status than those without diabetes (HR = 1.70, 95% CI: 1.18–2.46). Each 1% decrease in quadriceps cross‐sectional area was associated with an increased risk of WOMAC total score worsening (OR = 0.24, 95% CI: 0.17–0.33). Conversely, each 1% increase in quadriceps intramuscular fat was associated with a 38% increase in the risk of WOMAC total score worsening (OR = 1.38, 95% CI: 1.07–1.76). Additionally, each 1% decrease in quadriceps cross‐sectional area was associated with an increased risk of knee replacement within 9 years (HR = 0.70, 95% CI: 0.58–0.86).

#### 3.5.5 High‐IMFI Versus Low‐IMFI Groups

Raynauld et al. (2015) found that fat content in the vastus medialis (VM) was significantly higher in the high‐fat group than in the low‐fat group (SMD = 2.56, 95% CI: 2.12–3.00). When grouped by the median VM area (13 cm^2^), the larger‐area group had a significantly greater area than the smaller‐area group (SMD = 2.52, 95% CI: 2.08–2.95).

## 4 Discussion

This systematic review and meta‐analysis evaluated the IMFI‐KOA association, muscle‐specific patterns, and clinical prognostic value across 17 observational studies, with the goal of clarifying the overall effect size, identifying affected muscle groups, and synthesizing prognostic evidence.

The observed elevation of IMFI in KOA patients is biologically plausible. Adipose infiltration replaces contractile muscle fibers, directly compromising strength and power output. This is particularly detrimental to the quadriceps, which plays a critical role in dynamic knee stabilization; its dysfunction may alter joint loading and is associated with cartilage wear (Goodpaster et al. 2001; Zhao et al. 2026). Additionally, intramuscular adipose tissue secretes pro‐inflammatory adipokines that may promote synovial inflammation and matrix degradation (Andreea‐Ramona and Mirabela 2026; Goodpaster et al. 2023). However, these mechanistic interpretations are based on pathophysiological hypotheses derived from observational data, and the current evidence does not establish causality.

Substantial overall heterogeneity prompted subgroup analyses to clarify muscle‐specific patterns. At the aggregated anatomical level, IMFI in the anterior thigh muscles (rectus femoris, vastus lateralis, vastus medialis) demonstrated a significant association with KOA and was the primary driver of the overall effect. However, when analyzed individually, none of these three muscles reached statistical significance—a discrepancy likely attributable to inter‐study differences in measurement techniques, populations, and segmentation approaches (Garcia‐Diez et al. 2024; Kemnitz et al. 2017). At the individual muscle level, the strongest effect size was observed for the semimembranosus, followed by the adductor magnus/longus. This finding is biomechanically plausible: the semimembranosus is the primary internal rotator and knee flexor among the hamstrings and a key stabilizer of the posteromedial knee. Its fatty infiltration may weaken internal rotation and varus stability, potentially increasing medial compartment loading—the site most susceptible to KOA (Biltz et al. 2020; Chahla et al. 2021; Elias et al. 2009; Olewnik et al. 2025). The adductor magnus and adductor longus are involved in knee adduction and flexion and play an important role in frontal‐plane stability; fatty infiltration of these muscles may similarly affect joint mechanics (Bennell et al. 2007). In contrast, the sartorius and gracilis showed no significant associations, possibly due to their biarticular nature and strong compensatory capacity, or their relatively minor contribution to local knee mechanics (Schumacher et al. 2020; Zhang et al. 2020). Additionally, slow‐twitch fibers are more prone to fatty infiltration, and the semimembranosus contains a higher proportion of such fibers, further supporting site specificity (Gorski et al. 2024). However, it is crucial to emphasize that these findings originate from a single study (Jasinevicius et al. 2024) and require replication in larger, independent cohorts before any firm conclusions can be drawn. The preliminary nature of these observations must be consistently acknowledged throughout the interpretation.

Regarding imaging modalities, the CT subgroup demonstrated a significant pooled effect with moderate heterogeneity, whereas the MRI subgroup showed a non‐significant pooled effect with very high heterogeneity. This discrepancy should not be interpreted as evidence that CT is superior to MRI for IMFI assessment. Rather, it likely reflects: (1) the relatively small number of MRI studies included, limiting statistical power; (2) substantial heterogeneity in MRI sequences (T1‐weighted, Dixon, IDEAL) and quantification algorithms; (3) variability in measurement approaches (single‐slice vs. whole‐muscle); and (4) differences in segmentation methods (manual vs. semi‐automated) (Cotofana et al. 2010; Garcia‐Diez et al. 2024; Mohajer et al. 2022). CT attenuation values are relatively well standardized and reproducible, which explains the lower heterogeneity in the CT subgroup. However, MRI offers superior soft‐tissue contrast and the ability to quantify fat fraction more directly, and with standardization of protocols (e.g., Dixon techniques), it holds great promise for future research. Ultrasound was represented by only two effect sizes from a single study (J. Li et al. 2024), and its role in IMFI assessment remains to be established.

The prognostic value of IMFI extends across the KOA continuum. Elevated IMFI was consistently associated with reduced muscle strength, higher KOA incidence, structural progression, symptom worsening, and increased knee replacement risk. Notably, the magnitude of association with structural progression appears comparable to or greater than some traditional biomarkers. The differential associations of anterior versus posterior thigh IMFI with knee versus hip OA suggest potential joint‐specific and systemic effects. Collectively, these observations position IMFI as a potentially valuable research marker that requires further prospective validation before clinical application. However, these prognostic associations are derived from descriptive analyses and should be interpreted as hypothesis‐generating rather than confirmatory.

Notably, the association between IMFI and KOA appears to be independent of muscle volume, as evidenced by studies showing elevated IMFI despite preserved or even increased muscle cross‐sectional area. This underscores the importance of assessing muscle quality—not just quantity—in clinical evaluation.

The substantial overall heterogeneity (*I* ^2^ = 84.7%) warrants careful consideration. This level is expected given the clinical and methodological diversity across the 17 included studies. Key sources likely include: (1) clinical heterogeneity in participant characteristics, such as age (range 45–76 years), BMI (range 20.8–31.8 kg/m^2^), and KOA severity (KL grades 0–4), which influence baseline IMFI levels; (2) methodological heterogeneity in IMFI assessment, including different imaging modalities (CT, MRI, ultrasound), varied quantification metrics (fat fraction, attenuation values, echogenicity), and inconsistent muscle segmentation approaches; and (3) differences in study design (cross‐sectional vs. prospective) and control group definitions. Although factors such as age and BMI may contribute to the observed heterogeneity (Goodpaster et al. 2001, 2023), the primary driver appears to be the substantial methodological diversity across studies, particularly in imaging protocols and quantification thresholds. This underscores the need for standardized IMFI measurement protocols in future research.

This high level of heterogeneity has important implications for the certainty of our findings. According to GRADE considerations, the substantial inconsistency across studies reduces confidence in the pooled estimates. Moreover, the wide prediction interval (−0.21–1.47) indicates that the true effect in a future study could range from no association to a large positive effect, limiting the generalizability of our findings. Thus, while the meta‐analysis demonstrates a statistically significant association, the high heterogeneity and wide prediction interval suggest that the magnitude of this association may vary considerably across different populations and measurement protocols. This underscores the need for standardized IMFI measurement protocols in future research and cautions against overgeneralizing the pooled estimate to all clinical settings.

Compared with the previous meta‐analysis by Pedroso et al. (2019), which included 7 cross‐sectional studies and reported a significant but less detailed association, our updated review includes 17 studies with broader muscle group coverage, more diverse imaging modalities, and muscle‐specific subgroup analyses. The pooled SMD of 0.63 observed in our analysis is somewhat lower than the effect size reported in some earlier narrative syntheses, which may reflect the inclusion of more recent studies with larger sample sizes and more rigorous control group definitions, as well as our correction for within‐study non‐independence. Nevertheless, the overall direction and significance of the association remain consistent.

Limitations: Several limitations should be acknowledged. First, the predominance of cross‐sectional studies limits causal inferences, and the observational nature of all included studies precludes definitive conclusions about directionality. Second, definitions of high IMFI varied considerably across studies (median, quartiles, pre‐specified thresholds), and a universally accepted clinical cutoff is lacking. Third, most studies did not adjust for potential confounders, including physical activity, diet, and intramuscular inflammation. Fourth, sample sizes varied considerably, and small‐study effects may overestimate effect sizes. Fifth, most studies focused on anterior thigh muscles, with limited data on semimembranosus and adductors; although our subgroup analyses identify their associations, the evidence remains limited due to restricted raw data. Sixth, pooling diverse outcome measures (radiographic grading, MRI lesions, WOMAC scores, muscle strength) into a single SMD may have introduced additional heterogeneity. Seventh, while we addressed the issue of non‐independence by averaging within‐study effects for subgroup analyses, the limited number of studies precluded the use of more complex methods such as robust variance estimation. Eighth, we did not perform a formal GRADE assessment; given the predominance of observational studies and the substantial heterogeneity observed, the overall certainty of the evidence would likely be rated as moderate at best. This limits the strength of recommendations that can be derived from the current findings and underscores the need for future high‐quality prospective studies.

It is also important to consider alternative explanations for the observed associations. Aging, obesity, and pain‐related physical inactivity are all independently associated with both higher IMFI and KOA, and may confound the relationship. Furthermore, reverse causation cannot be excluded: KOA‐related pain and functional limitation may lead to reduced physical activity and subsequent muscle disuse, which in turn promotes fatty infiltration. The cross‐sectional design of most included studies precludes disentangling these temporal relationships. Future prospective studies with repeated measures of IMFI and physical activity are needed to clarify the directionality of these associations.

Future directions: Standardizing MRI Dixon techniques for IMFI quantification, conducting large‐scale prospective cohorts, establishing clinical cutoff values, and designing interventional trials to evaluate IMFI reduction for KOA prevention are priorities. Integrating muscle biopsy and multi‐omics approaches will further elucidate the underlying mechanisms.

## 5 Conclusion

This meta‐analysis of observational data demonstrates a positive association between IMFI and KOA though causality cannot be inferred. At the aggregated anatomical level, anterior thigh muscles show the strongest association, whereas individual muscle findings (e.g., semimembranosus) are based on limited evidence and require replication. Imaging modality comparisons suggest that CT provides more standardized measurements than MRI in current studies, but this reflects methodological heterogeneity rather than inherent superiority. Elevated IMFI is consistently associated with adverse outcomes across the KOA continuum in individual studies, suggesting that IMFI may hold promise as a prognostic indicator in research settings, though clinical translation requires further prospective validation; however, these findings are derived from observational data and require confirmation in future prospective studies. Future research should prioritize standardized MRI protocols, large‐scale prospective cohorts, validated clinical cutoff values, and interventional trials to determine whether reducing IMFI modifies disease progression.

## 6 Implications of Physiotherapy Practice

The findings of this systematic review and meta‐analysis have potential implications for physiotherapy practice in KOA management, though they should be interpreted with caution given the observational nature of the evidence and the substantial heterogeneity observed across studies. First, given that IMFI is independently associated with reduced muscle strength and poorer clinical outcomes in individual studies, clinicians should be aware that muscle quality—not merely muscle quantity (e.g., cross‐sectional area or volume)—may be an important determinant of function. However, routine assessment of IMFI in standard physiotherapy practice is not currently recommended. This is due to the limited availability of advanced imaging modalities (MRI, CT, point‐of‐care ultrasound) in most clinical settings, the lack of validated clinical cutoff values, and the absence of evidence that IMFI‐guided interventions improve patient outcomes. In settings where imaging resources are available and clinically indicated, they may serve as adjunctive tools to assess fatty changes in key muscles such as the rectus femoris, but this remains a research application rather than a clinical standard. Second, the robust association observed in the anterior thigh muscle group (rectus femoris, vastus lateralis, and vastus medialis), along with preliminary signals from the semimembranosus, supports the continued emphasis on targeted, progressive resistance training. Physiotherapy protocols may consider high‐intensity strengthening exercises for both the quadriceps and hamstrings to address the functional consequences of fatty infiltration. Third, although elevated IMFI appears to be associated with symptom worsening and knee replacement risk, routine risk stratification based on IMFI is not yet feasible in clinical practice. Physiotherapists can instead utilize indirect clinical indicators—such as reduced specific torque, altered muscle stiffness/elasticity, and poor functional performance—to identify high‐risk patients and tailor individualized interventions, including neuromuscular electrical stimulation or adjunctive metabolic strategies (e.g., weight management), alongside conventional exercise therapy. Future research should focus on validating clinically accessible and cost‐effective IMFI assessment methods, establishing standardized protocols, and demonstrating that IMFI‐guided interventions improve meaningful clinical outcomes before routine integration of muscle quality monitoring into physiotherapy practice can be recommended.

## Author Contributions

**Lili Yu:** conceptualization, methodology, literature search, study selection, data extraction, quality assessment, formal analysis, visualization, writing – original draft. **Cong Chen:** study selection, data extraction, quality assessment, formal analysis. **Guancong Zhang:** study selection, data extraction, quality assessment. **Qiuchen Huang:** methodology, formal analysis, writing – review and editing. **Miao Ye:** study selection, data extraction, quality assessment, writing – review and editing. **Yukiko Makihara:** conceptualization, writing – review and editing, supervision. **Yusuke Nishida:** conceptualization, methodology, writing – review and editing, supervision. All authors have read and approved the final manuscript.

## Funding

The authors have nothing to report.

## Ethics Statement

The authors have nothing to report.

## Consent

The authors have nothing to report.

## Conflicts of Interest

The authors declare no conflicts of interest.

## Supporting information


Supporting Information S1

Supporting Information S2

## Data Availability

The datasets supporting the conclusions of this meta‐analysis are included in the article and its supplementary files. Search strategies and data extraction forms are available from the corresponding author upon reasonable request.
