# Supplementary material for: Intramuscular Fat Infiltration and Knee Osteoarthritis: A Systematic Review and Meta‐Analysis
Source: Physiother Res Int. 2026 Aug 26;31(4):e70330. doi: 10.1002/pri.70330 (PMC13519057; doi:10.1002/pri.70330)
Supplement: Supplementary file 2 [file PRI-31-e70330-s002.docx]

**Table S1** Detailed Search Strategies for Each Database

| Database | Query formulation | Number of literatures detected | Date of search |
| --- | --- | --- | --- |
| PubMed | #1: (((((“Osteoarthritis, Knee”[Mesh]) OR (“Osteoarthritis”[Mesh])) OR (osteoarthr)) OR (arthrosis)) OR (gonarthr)) OR (degenerative arthriti) #2: (#1) AND (knee) #3: (((((((((“Adipose tissue”[Mesh]) OR (“myosteatosis”)) OR (“Intramuscular fat”)) OR (Muscle fat infiltration)) OR (Intermuscular fat)) OR (Intermuscular adipose tissue)) OR (Intramuscular adipose tissue)) OR (Intramyocellular lipids)) OR (Muscles fat)) OR (Muscle lipid) #4: (((((((((((((((((((((((((((((“incidence”[Mesh]) OR (“Muscle Strength”[Mesh])) OR (“Inflammation”[Mesh])) OR (“cartilage”[Mesh])) OR (“meniscus”[Mesh])) OR (“bone marrow”[Mesh])) OR (“biomarkers”[Mesh])) OR (“physical functional performance”[Mesh])) OR (incidence)) OR (inciden)) OR (cartilage)) OR (cartilage)) OR (meniscus)) OR (menisci)) OR (bone marrow lesions)) OR (degeneration)) OR (degenerat)) OR (physical performance)) OR (physical functional performance)) OR (clinical severity)) OR (sever)) OR (function)) OR (function)) OR (performance)) OR (perform)) OR (inflammation)) OR (inflammat)) OR (biomarkers)) OR (biomarker) #5: ((“cohort studies”[Mesh]) OR (Cohort study)) OR (Observation*) #6: (#2) AND (#3) AND (#4) AND (#5) | 169 | 2025-12-15 |
| Web of Science | #1: (((((TS=("Osteoarthritis, Knee")) OR TS=("knee osteoarthritis")) OR TS=(osteoarthr*)) OR TS=(arthrosis*)) OR TS=(gonarthr*)) OR TS=(degenerative arthriti*) #2: (((((((TS=("myosteatosis")) OR TS=("Intramuscular fat")) OR TS=(Muscle fat infiltration)) OR TS=(Intermuscular fat )) OR TS=(Intermuscular adipose tissue )) OR TS=(Intramuscular adipose tissue )) OR TS=(Intramyocellular lipids Muscles fat )) OR TS=(Muscle lipid) #3: (((((((((((((((((((TS=(incidence)) OR TS=(inciden*)) OR TS=(Cartilage)) OR TS=(cartilage*)) OR TS=(Meniscus)) OR TS=(menisci)) OR TS=(Bone Marrow Lesions)) OR TS=(Degeneration)) OR TS=(degenerat*)) OR TS=(physical performance)) OR TS=(physical functional performance)) OR TS=(clinical severity)) OR TS=(sever*)) OR TS=(function)) OR TS=(function*)) OR TS=(performance)) OR TS=(perform*)) OR TS=(inflammation)) OR TS=(Inflammat* )) OR TS=(biomarkers )) OR TS=(biomarker*) #4: (TS=(cohort study)) OR TS=(observation*) #5: #1 AND #2 AND #3 AND #4 and Preprint Citation Index (Exclude – Database) | 62 | 2025-12-15 |
| Scopus | ( TITLE-ABS-KEY ( cohort study ) OR TITLE-ABS-KEY ( observation* ) ) AND ( TITLE-ABS-KEY ( incidence ) OR TITLE-ABS-KEY ( inciden* ) OR TITLE-ABS-KEY ( Cartilage ) OR TITLE-ABS-KEY ( cartilage* ) OR TITLE-ABS-KEY ( Meniscus ) OR TITLE-ABS-KEY ( menisci ) OR TITLE-ABS-KEY ( Bone Marrow Lesions ) OR TITLE-ABS-KEY ( Degeneration ) OR TITLE-ABS-KEY ( degenerat* ) OR TITLE-ABS-KEY ( physical performance ) OR TITLE-ABS-KEY ( physical functional performance ) OR TITLE-ABS-KEY ( clinical severity ) OR TITLE-ABS-KEY ( sever* ) OR TITLE-ABS-KEY ( function ) OR TITLE-ABS-KEY ( function* ) OR TITLE-ABS-KEY ( performance ) OR TITLE-ABS-KEY ( perform* ) OR TITLE-ABS-KEY ( inflammation ) OR TITLE-ABS-KEY ( Inflammat* ) OR TITLE-ABS-KEY ( biomarkers ) OR TITLE-ABS-KEY ( biomarker* ) ) AND ( TITLE-ABS-KEY ( "myosteatosis" ) OR TITLE-ABS-KEY ( "Intramuscular fat" ) OR TITLE-ABS-KEY ( Muscle fat infiltration ) OR TITLE-ABS-KEY ( Intermuscular fat ) OR TITLE-ABS-KEY ( Intermuscular adipose tissue ) OR TITLE-ABS-KEY ( Intramuscular adipose tissue ) OR TITLE-ABS-KEY ( Intramyocellular lipids ) OR TITLE-ABS-KEY ( Muscle lipid ) OR TITLE-ABS-KEY ( Muscles fat ) ) AND ( TITLE-ABS-KEY ( knee ) ) AND ( TITLE-ABS-KEY ( "knee osteoarthritis" ) OR TITLE-ABS-KEY ( osteoarthr* ) OR TITLE-ABS-KEY ( arthrosis* ) OR TITLE-ABS-KEY ( gonarthr* ) OR TITLE-ABS-KEY ( degenerative arthriti* ) ) | 77 | 2025-12-15 |
| Embase | #1: 'knee osteoarthritis'/exp OR 'knee osteoarthritis' OR 'osteoarthritis'/exp OR 'osteoarthritis' OR osteoarthr*:ti,ab,kw OR arthrosis*:ti,ab,kw OR gonarthr*:ti,ab,kw OR 'degenerative arthriti*':ti,ab,kw #2: 'intramuscular fat'/exp OR 'intermuscular fat'/exp OR 'adipose tissue'/exp OR 'myosteatosis':ti,ab,kw OR 'intramuscular fat':ti,ab,kw OR 'muscle fat infiltration':ti,ab,kw OR 'intermuscular fat':ti,ab,kw OR 'intermuscular adipose tissue':ti,ab,kw OR 'intramuscular adipose tissue':ti,ab,kw OR 'intramyocellular lipids':ti,ab,kw OR 'muscles fat':ti,ab,kw OR 'muscle lipid':ti,ab,kw #3: 'incidence'/exp OR 'inflammation'/exp OR 'muscle strength'/exp OR 'cartilage'/exp OR 'knee meniscus'/exp OR 'bone marrow'/exp OR 'biological marker'/exp OR 'physical performance'/exp OR incidence:ti,ab,kw OR inciden*:ti,ab,kw OR cartilage:ti,ab,kw OR cartilage*:ti,ab,kw OR meniscus:ti,ab,kw OR menisci:ti,ab,kw OR 'bone marrow lesions':ti,ab,kw OR degeneration:ti,ab,kw OR degenerat*:ti,ab,kw OR 'physical performance':ti,ab,kw OR 'physical functional performance':ti,ab,kw OR 'clinical severity':ti,ab,kw OR sever*:ti,ab,kw OR function:ti,ab,kw OR function*:ti,ab,kw OR performance:ti,ab,kw OR perform*:ti,ab,kw OR inflammation:ti,ab,kw OR inflammat*:ti,ab,kw OR biomarkers:ti,ab,kw OR biomarker*:ti,ab,kw #4: 'cohort analysis'/exp OR cohort:ti,ab,kw OR observation*:ti,ab,kw #5: knee:ti,ab,kw #6: #1 AND #2 AND #3 AND #4 AND #5 | 229 | 2025-12-15 |

**Table S2** Basic Characteristics of the Included Studies

| First author, year | IMFI measurement method | Muscle site measured | Main IMFI quantitative indicators | Definition of high/low grouping |
| --- | --- | --- | --- | --- |
| Beattie, 2012 | MRI T1 | right thigh quadriceps | quadriceps volume (cm³), intermuscular fat volume (cm³) | ROA vs. non-ROA (2-year change) |
| Blumenfeld, 2013 | DXA | both lower limbs | lower limb fat mass (kg), lower limb lean mass (kg), abdominal fat mass (kg) | K/L ≥ 2 vs. normal at 10 years |
| Conroy, 2012 | CT | total thigh muscle, quadriceps | muscle area, attenuation (HU), intermuscular fat | RKOA group vs. non-RKOA group |
| Distefano, 2024 | MRI + muscle biopsy | anterior thigh | muscle fat infiltration (%), OXPHOS | KL 3–4 vs. KL 0–1 |
| Jasinevicius, 2024 | CT | rectus femoris, vastus lateralis, semitendinosus, etc. | muscle attenuation value (HU) | young OA vs. young healthy |
| Kemnitz, 2018 | MRI (single slice) | knee extensors, knee flexors | muscle ACSA, intermuscular fat ACSA | symptom progressors vs. non-progressors |
| Kitamura, 2025 | non-contrast CT | gluteal muscles, quadriceps, calf | HU classification, CSA ratio | affected side vs. healthy side |
| Kumar, 2021 | MRI IDEAL water-fat separation | quadriceps, vastus medialis | intramuscular fat fraction (%) | continuous variable (per 1% increase) |
| Lee, 2025 | CT + AI segmentation | anterior/medial/posterior thigh, gluteal muscles | muscle volume, intramuscular fat proportion (%) | more painful side vs. contralateral side |
| Li, 2024 | ultrasound + Myoton PRO | rectus femoris, vastus medialis, vastus lateralis | echo intensity (0–255), shear modulus, muscle tone, stiffness | affected side vs. healthy side; affected side vs. healthy controls |
| Manatrakul, 2024 | MRI T1 | quadriceps, hamstrings, adductors | muscle volume (cm³), intermuscular fat, subcutaneous fat | by sex / by RKOA |
| Mohajer, 2022 (DM) | MRI deep learning | quadriceps, flexors, adductors | intramuscular fat CSA, contractile ratio (%) | DM vs. non-DM (with KOA) |
| Mohajer, 2022 (KOA) | MRI deep learning | quadriceps, flexors, adductors | muscle CSA, intramuscular fat CSA | KOA group vs. non-KOA group |
| Raynauld, 2015 | MRI T1 | vastus medialis (VM) | VM fat (%), VM area (cm²) | high/low divided by median of 5.1% |
| Taniguchi, 2023 | MRI T1 + Dixon | quadriceps (RF, VI, VL, VM) | intramuscular fat (%), muscle volume (cm³) | early KOA vs. healthy controls |
| Teoli, 2022 | MRI T1 | vastus medialis (VM) | VM fat (%), VM area (mm²) | KOA group vs. healthy controls |
| Weng, 2025 | MRI 1.5T Dixon | anterior + posterior thigh | muscle fat fraction (%) | Q4 vs. Q1 |

Note: ACSA = Anatomical Cross-Sectional Area; CSA = Cross-Sectional Area; VM = Vastus medialis; RF = Rectus femoris; VI = Vastus intermedius; VL = Vastus lateralis; OXPHOS = Oxidative phosphorylation.

**Table S3** Summary of the Risk of Bias Assessment

| First author, year | Study design | Assessment tool | Total score | Quality grade |
| --- | --- | --- | --- | --- |
| Weng, 2025 | prospective cohort | NOS | 9 | high |
| Blumenfeld, 2013 | prospective cohort | NOS | 8 | high |
| Raynauld, 2015 | RCT longitudinal | NOS | 9 | high |
| Mohajer, 2023 (DM) | prospective cohort | NOS | 9 | high |
| Mohajer, 2022 (KOA) | prospective cohort | NOS | 9 | high |
| Kemnitz, 2018 | prospective cohort | NOS | 9 | high |
| Beattie, 2012 | prospective cohort | NOS | 7 | moderate |
| Kumar, 2021 | prospective cohort | NOS | 7 | moderate |
| Kitamura, 2025 | cross-sectional | JBI | 8 | high |
| Li, 2024 | cross-sectional | JBI | 8 | high |
| Conroy, 2012 | cross-sectional | JBI | 8 | high |
| Distefano, 2024 | cross-sectional | JBI | 8 | high |
| Taniguchi, 2023 | cross-sectional | JBI | 8 | high |
| Manatrakul, 2024 | cross-sectional | JBI | 8 | high |
| Teoli, 2022 | cross-sectional | JBI | 8 | high |
| Jasinevicius, 2024 | cross-sectional | JBI | 7 | moderate |
| Lee, 2025 | cross-sectional | JBI | 7 | moderate |

**Figure S1** Forest plot of anatomical region subgroups associated with IMFI and KOA

Note: Stratified analysis of the association between IMFI and KOA by anatomical region (anterior muscle group, posterior muscle group, medial muscle group, and composite index).

**Figure S2** Forest plot by measurement method subgroup

Note: Stratified analysis of CT, ultrasound, and MRI based on the IMFI measurement method
